# Physical Activity in Adolescents and Young Adults with Cerebral Palsy

**DOI:** 10.1155/2017/8080473

**Published:** 2017-12-20

**Authors:** Lisa Waltersson, Elisabet Rodby-Bousquet

**Affiliations:** ^1^Eskilstuna Habilitation Centre, Eskilstuna, Sweden; ^2^Centre for Clinical Research, Uppsala University, Region Västmanland, Västerås, Sweden; ^3^Department of Clinical Sciences, Lund, Orthopaedics, Lund University, Lund, Sweden

## Abstract

The aim of this study was to examine the level of physical activity in adults with cerebral palsy (CP) and to analyse its relationship with physical activity as adolescents, pain, and gross motor function. A prospective cohort study was performed using data from the Swedish National CP Registry (CPUP) for all 129 individuals born in 1991–1993 living in Skåne and Blekinge who reported to CPUP at 14–16 years of age. Physical activity as adult was analysed relative to physical activity as adolescents, pain, and the Gross Motor Function Classification System (GMFCS). Seventy-one individuals at GMFCS I–V were followed up as adults and included in the analyses. Of these, 65% were physically active, but only 56% performed physical activity at least once a week. Their physical activity as adults differed relative to their physical activity as adolescents (*p* = 0.011) but not to pain or GMFCS. Being physically active as an adolescent doubled the probability of being active as an adult (OR 2.1; *p* = 0.054), indicating that physical activity in adults with CP is related to their physical activity as adolescents. Therefore, interventions to increase physical activity among adolescents with CP are likely also to improve physical activity in adulthood.

## 1. Introduction

Although the brain lesions causing cerebral palsy (CP) are nonprogressive, the symptoms change over time as a child grows and ages [1]. Many adults with CP experience a decrease in gross motor function, and 10% stop walking before 35 years of age [2–4]. The decrease in locomotion skills is associated with fatigue, pain, impaired balance, and limitations on the ability to participate in personally adjusted physical activity [3, 4]. About 40% of adults with CP report pain regularly. The pain is often located to joints with limited range of motion and the most common pain location is back and hip [5]. Both adolescents and adults with CP are less physically active than their typically developed peers and often do not achieve the recommended guidelines for physical activity [6, 7]. Adults with bilateral CP have a reduced level of everyday physical activity and reduced aerobic fitness compared with nondisabled adults [8]. A reduced level of gross motor function is associated with lower participation in physical activities [9, 10].

Physical activity, defined as “any bodily movement produced by skeletal muscles that requires energy expenditure” [11, 12], leads to a reduced risk of cardiovascular disease and overweight [13], has a positive effect on self-image and mental health [14, 15], and results in a higher bone mass density in children and adults [16].

The recommendations regarding physical activity for adults are moderate-to-high-intensity physical activity every week. Moderate physical activity for an hour a day is recommended to further enhance the health benefits of physical activity, and moderate aerobic exercise should be combined with strength and flexibility exercises at least twice a week [17]. Children and adolescents are recommended to be physically active for at least an hour a day with diversified activities, including fitness, strength, coordination, and flexibility exercises.

Because of the positive effects of physical activity, it is of interest to study the level of physical activity in young adults with CP after their transition from paediatric rehabilitation units to adult health care and the extent to which their physical activity in adulthood can be predicted by their level of physical activity as an adolescent, their pain, and their level of gross motor function. In this study, the term “physical activity” refers to all physical activities that lead to increased energy consumption, including moderate to vigorous physical efforts such as walking, swimming, soccer, dancing, weightlifting, cycling, and horseback riding.

The aim of the study was to examine the level of physical activity in young adults with CP and to analyse the extent to which this can be predicted by their physical activity as adolescents, their pain, and their gross motor function.

## 2. Materials and Methods

We performed a prospective cohort study using registry data from the Swedish National Quality Registry for individuals with CP (CPUP). CPUP is a combined registry and surveillance programme for children and adults with CP in Scandinavian countries [18]. In Sweden, over 95% of all children with CP are enrolled in CPUP. Clinical and radiographic examinations are performed regularly based on the child's age and gross motor function according to a standardised assessment form and manual, and the data are reported to the web-based registry.

### 2.1. Participants

All 129 individuals with CP born in 1991–1993 living in the counties of Skåne and Blekinge who were reported to the CPUP registry at the age of 14–16 years were included in the study at baseline. These individuals were chosen because they were followed up by CPUP from its initiation in 1994, were likely to have transitioned from paediatric rehabilitation units into adult health care, and were likely to have had at least one examination as adults within the follow-up programme. Individuals for whom an examination as adults was not recorded in CPUP or who lacked data reported for physical activity as adults were excluded from the analyses.

### 2.2. Data Collection

Data were extracted at three separate time points: at baseline when the individuals were 14–16 years old, when they were 17-18 years old, and as adults at 19–22 years of age. If the individual had reports from more than one examination during the age range of 14–16 or 17-18 years, data from the earliest reports were included. For adults with 19–22 years of age, the most recent report was used. These age groups were chosen to allow analysis of the participants' level of physical activity at Swedish secondary school and upper secondary school and as adults after transition from paediatric and adolescent rehabilitation units.

### 2.3. Classifications and Measurements

The variables analysed were age at assessment, sex, physical activity, pain, and classification level using the expanded and revised version of the Gross Motor Function Classification System (GMFCS) [19]. All assessments were performed by local physiotherapists in a standardised manner employing an assessment form and an accompanying manual (http://www.cpup.se). GMFCS level was assessed by the physiotherapist. Pain was reported by the individual or by proxy as whether or not the individual was in pain (yes/no). Physical activity was reported by the individual or by proxy. For school-aged children, physical activity was reported both for leisure time and for participation in physical education at school. For adults, it was reported as physical activity at any time during the week. Physical activity for children was reported using the following questions: (1) “Has the person actively participated in/performed physical activities/sports in school/preschool since the last assessment?” (2) “Has the person participated in/performed physical leisure activities/sports since the last assessment?” For adults, the question was the following: “Has the person participated in/performed physical activities/sports regularly since the last assessment?” The options reported by the person or by proxy were (A) no; (B) yes, less than once a week; (C) yes, 1 to 2 times a week; or (D) yes, 3 to 5 times a week.

### 2.4. Statistical Analyses

Binary logistic regression was used to estimate the relationship of physical activity in adults with physical activity as an adolescent, pain, and GMFCS level [20]. The results are presented as the odds ratio (OR) with a 95% confidence interval and *p* values. The OR can be explained as the ratio between two odds which expresses the probability of an event occurring. In the binary logistic regression, physical activity was defined as “yes” if they were physically active at least once a week and as “no” if they were not. For all other analyses, physical activity in secondary school and upper secondary school was treated as “no physical activity,” “physical activity in either school or in leisure time,” or “physical activities in both school and leisure time.” For children reported to be physically active in both settings, data were summed up to present overall physical activity in times per week. Fisher's exact test was used to analyse differences in physical activity as an adult in relation to GMFCS level, pain, and physical activity as an adolescent. All statistical tests were two-sided and *p* values below the standard value of 0.05 were considered significant. IBM SPSS Statistics 22 was used to perform the statistical analyses.

Ethical approval was granted by the Medical Ethics Committee at Lund University and the study was conducted in accordance with the Declaration of Helsinki (1964). Permission to extract data was obtained from the registry holder.

## 3. Results

**Figure 1:**
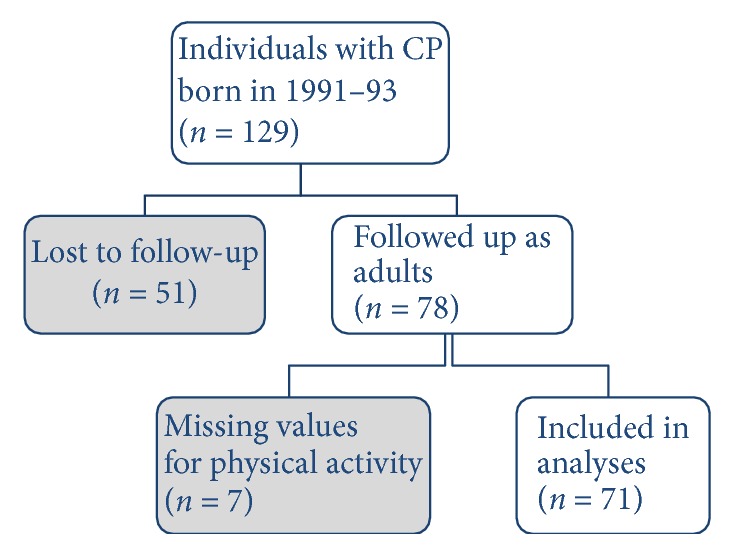
Flowchart showing included participants, drop-outs, and missing values.

A total of 129 individuals born in 1991–1993 had at least one examination reported to the CPUP registry between the ages of 14–16 years. Of these, 51 individuals were lost to follow-up and had no data reported as adults and were therefore excluded from the analyses. For seven other individuals, data regarding physical activity as adults were missing (Figure 1). The remaining 71 individuals (45 females) at GMFCS I (*n* = 22), II (*n* = 16), III (*n* = 10), IV (*n* = 14), and V (*n* = 9) were included in the analyses. There was a higher proportion of adults at GMFCS I and of men in the nonparticipant group compared with the participant group (Table 1).

Of the 71 participants, 46 (65%) reported that they participated in physical activities as adults but only 56% were active on a regular basis, that is, at least once a week (Table 2). Differences in physical activity were seen between the participants at the different GMFCS levels, but no significant differences were found for the number of times per week participants performed physical activity (*p* = 0.779) or for weekly participation in activities (*p* = 0.351). Individuals at GMFCS levels II and III were most physically active, with respect to both performing physical activity and participating in that activity at least weekly. The individuals at GMFCS level IV were least physically active, with 43% participating weekly. Pain was reported by 30 individuals (42%, with no significant difference between GMFCS levels; *p* = 0.484). A higher proportion (58%) of individuals who were not physically active reported pain. The reverse was seen for individuals who participated in a physical activity 3–5 times per week, where a higher percentage reported that they were pain-free (68%) (Table 2).

There was a significant difference (*p* = 0.011) in physical activity as an adult associated with previous physical activity in school or leisure time (Table 3). Of the adolescents aged 14–16 years, 6% did not engage in physical activity in their leisure time or participate in physical education at school while attending Swedish secondary school. This number increased to 18% at the age of 17-18 years while attending upper secondary school. The proportion of participants who engaged in physical activity in their leisure time and also participated in physical education in school decreased from 44% in Swedish secondary school to 41% in upper secondary school. Of the adolescents who did not participate in physical education and did not engage in physical activity in their leisure time, 31% engaged in physical activity at least once a week as adults. Of the participants who participated in school physical education and engaged in physical activity in their leisure time during upper secondary school, 68% participated in regular physical activity at least once a week as adults (Table 3).

Being physically active in leisure time and in physical education at upper secondary school as an adolescent seemed to double the probability of being physically active as an adult (OR 2.1; *p* = 0.054). Pain and GMFCS level did not affect the probability of being physically active as an adult (Table 4).

## 4. Discussion

This study examined the level of physical activity in young adults with CP and analysed the extent to which this could be explained by their physical activity as an adolescent, the occurrence of pain, and their GMFCS level. The results revealed that 43% of the participants were not physically active on a regular basis, that is, at least once a week. This finding suggests that adults with CP are less physically active compared with the general population and indicates that many are not physically active to the extent required to meet general recommendations. This result is consistent with those of earlier research which showed that adults with CP may not participate in physical activities to the extent required to experience the associated health benefits [21].

In this study, no significant association between physical activity and GMFCS level was found, and the GMFCS level did not affect the probability of being physically active as an adult. This result is in conflict with previous research that indicated that a higher GMFCS level was associated with lower participation in physical activities [9, 10]. Our result is surprising and may suggest that the needs of adults with greater physical disabilities are being well met by caregivers, families, and society and that resources in terms of personal assistance and time are provided to allow physical activity.

Pain was reported by 42% of participants, which is consistent with previous research [5]. A higher proportion of participants without pain were physically active compared with those who experienced pain, and although pain appeared to reduce the probability of being physically active, this reduction was not significant. This could partially be explained by the small number of participants. However, previous research has found that adults with CP only let pain interfere to a limited extent with their participation in activities [5].

There was a difference in physical activity as an adult depending on previous physical activity as an adolescent and physical activity as an adolescent doubled the probability of being physically active as an adult. The authors of previous studies have discussed the importance of interventions for individuals with CP during adolescence and when the individual is a young adult because of their strong influence on the adult lifestyle [22]. According to Hallal et al. [23], physical activity in adolescents contributes to a healthier and more physically active lifestyle as adult. The findings of our study show that this relationship also applies to adolescents and adults with CP. Although several studies have shown the benefits of a physically active lifestyle as an adolescent, there are no guidelines for health-promoting interventions during the transition of adolescents with CP into adult health care [24].

A relatively high proportion of the participants in this study did not participate in physical education during secondary school. This is consistent with previous research showing that 13% of children and adolescents with CP did not participate in physical education at school and that half of the children were not physically active in their leisure time [25]. Motivation for being physically active and having the opportunity to learn the rules and procedures of different games and sports are an important part of a school's physical education programme [26] and also the most important factor explaining physical activity in adults with disabilities [27, 28]. Adolescents will not attain this knowledge unless they participate in school-based physical education [29].

Previous research studying physical activity among children and adolescents in the same region of Sweden found a different sex distribution compared with this study [25]. Because the previous study found no difference in physical activity between the sexes, the different distribution in this study is unlikely to have affected the outcome. However, this raises interesting and important questions regarding whether women were more likely than men to continue their enrolment in a surveillance programme such as CPUP as adults and, if so, why.

The results of this study in combination with those of earlier studies show that time and resources spent on motivating adolescents with CP and helping them find a physical activity that they enjoy might increase the probability of a more physically active lifestyle as young adults. This could improve their health and therefore reduce their need for health care later in life. Spending time and resources to physically activate adolescents with CP could therefore be socioeconomically justifiable.

There are some limitations to this study. The fact that many of the analyses failed to reach statistical significance might be because of the small number of participants in the study. The relatively large drop-out rate in this study could be explained by the fact that data from only two regions in Sweden were included. This means that any participant who moved to another county or abroad was lost to follow-up and therefore was excluded from the study. The selected age range for follow up was 19–22 years; it would not be surprising if individuals who lived in the catchment area at the age of 14–16 years had moved at the time of follow-up, because this is a period in life where individuals are likely to move to find a job or for studies. The high number of individuals at GMFCS I in the drop-outs may be because individuals with CP who have a high level of motor function are less likely to experience limitations regarding participation and activity [9, 10] and therefore end their contact with the rehabilitation team and also choose to end their participation in the CPUP registry. Because individuals with a lower GMFCS level are more likely to be physically active [9, 10], the GMFCS distribution of the drop-outs may have led to a lower estimate of the overall physical activity of participants in this study. Another limitation is that physical activity is only reported as times per week and there is no data of duration or intensity.

A strength of this study was the use of the National Quality Registry CPUP. By using a National Quality Registry, all children and adults invited to participate in CPUP could be part of the study. This means that the study has high external validity because the included participants can be considered an adequate representation of the total population. Therefore, the results from this study can probably be generalized to similar countries.

## 5. Conclusions

Slightly more than half of the adults with CP perform a physical activity at least once a week. Being physically active as an adolescent with CP seems to increase the likelihood of being physically active as an adult. Therefore, interventions to increase physical activity among adolescents with CP are recommended.

## Figures and Tables

**Table 1:** The total population of persons with CP born in 1991–93 living in Skåne and Blekinge and with a CPUP-recorded examination carried out between the ages of 14 and 16 years—participants and nonparticipants.

Characteristics		All *n* = 129 *n* (%)	Participants *n* = 71 *n* (%)	Nonparticipants *n* = 58 *n* (%)
Sex	Female	72 (56%)	45 (63%)	27 (47%)
	Male	57 (44%)	26 (37%)	31 (53%)
Region	Skåne	116 (90%)	61 (86%)	55 (95%)
	Blekinge	13 (10%)	10 (14%)	3 (5%)
GMFCS	I	58 (46%)	22 (31%)	36 (62%)
	II	24 (19%)	16 (23%)	8 (14%)
	III	12 (9%)	10 (14%)	2 (3%)
	IV	19 (15%)	14 (20%)	5 (7%)
	V	16 (12%)	9 (13%)	7 (12%)

*Note*. GMFCS, Gross Motor Function Classification System.

**Table 2:** Physical activity as adult relative to GMFCS level and pain.

Physical activity as an adult	GMFCS					Pain	
	I	II	III	IV	V	No	Yes
	*n* = 22	*n* = 16	*n* = 10	*n* = 14	*n* = 9	*n* = 37	*n* = 30
	*n* (%)	*n* (%)	*n* (%)	*n* (%)	*n* (%)	*n* (%)	*n* (%)
No	8 (36)	3 (19)	3 (30)	7 (50)	4 (44)	10 (27)	14 (47)
<1 time/week	3 (14)	2 (13)	0 (0)	1 (7)	0 (0)	3 (8)	3 (10)
1-2 times/week	4 (18)	5 (31)	4 (40)	1 (7)	2 (22)	9 (24)	6 (20)
3–5 times/week	7 (32)	6 (38)	3 (30)	5 (36)	3 (33)	15 (41)	7 (23)

*Note*. GMFCS, Gross Motor Function Classification System.

**Table 3:** Physical activity as an adult relative to physical activity as an adolescent in secondary school (14–16 years) and upper secondary school (17-18 years).

Physical activity as an adult	Physical activity, 14–16 years			Physical activity, 17-18 years		
	No	School or leisure time	School and leisure time	No	School or leisure time	School and leisure time
	*n* = 4	*n* = 35	*n* = 31	*n* = 13	*n* = 29	*n* = 29
	*n* (%)	*n* (%)	*n* (%)	*n* (%)	*n* (%)	*n* (%)
No	2 (50)	15 (43)	7 (23)	9 (69)	12 (41)	4 (14)
<1 time/week	0 (0)	1 (3)	5 (16)	0 (0)	1 (3)	5 (17)
1-2 times/week	0 (0)	7 (20)	9 (29)	1 (8)	5 (17)	10 (34)
3–5 times/week	2 (50)	12 (34)	10 (32)	3 (23)	11 (38)	10 (34)

**Table 4:** The odds ratio (OR) with 95% confidence interval (CI) of previous physical activity at 17-18 years, pain, and GMFCS level predicting the physical activity as an adult of an individual with CP. Physical activity as adolescent and GMFCS were treated as continuous variables and no pain was used as reference category.

	OR	95% CI	*p*
Physical activity as an adolescent^*∗*^	2.1	(1.0–4.5)	0.054
Pain	0.5	(0.2–1.3)	0.156
GMFCS	1.1	(0.7–1.6)	0.734

*Note*. GMFCS, Gross Motor Function Classification System; ^*∗*^during upper secondary school.
